# Effect of cinnamon on gastric emptying, arterial stiffness, postprandial lipemia, glycemia, and appetite responses to high-fat breakfast

**DOI:** 10.1186/1475-2840-10-78

**Published:** 2011-09-07

**Authors:** Oonagh Markey, Conor M McClean, Paul Medlow, Gareth W Davison, Tom R Trinick, Ellie Duly, Amir Shafat

**Affiliations:** 1 Department of Physical Education & Sport Sciences, University of Limerick, Limerick, Ireland; 2 Sport and Exercise Science Research Institute, University of Ulster, Belfast, Northern Ireland BT37 0QB, UK; 3 Ulster Hospital, Dundonald, Belfast, Northern Ireland BT16 1RH, UK; 4 Department of Physiology, National University of Ireland, Galway, Galway, Ireland

**Keywords:** gastrointestinal, antioxidant capacity, obesity, type 2 diabetes, polyphenols, fatty acids, omega-3 fatty acids

## Abstract

**Background:**

Cinnamon has been shown to delay gastric emptying of a high-carbohydrate meal and reduce postprandial glycemia in healthy adults. However, it is dietary fat which is implicated in the etiology and is associated with obesity, type 2 diabetes and cardiovascular disease. We aimed to determine the effect of 3 g cinnamon (*Cinnamomum zeylanicum*) on GE, postprandial lipemic and glycemic responses, oxidative stress, arterial stiffness, as well as appetite sensations and subsequent food intake following a high-fat meal.

**Methods:**

A single-blind randomized crossover study assessed nine healthy, young subjects. GE rate of a high-fat meal supplemented with 3 g cinnamon or placebo was determined using the ^13^C octanoic acid breath test. Breath, blood samples and subjective appetite ratings were collected in the fasted and during the 360 min postprandial period, followed by an *ad libitum *buffet meal. Gastric emptying and 1-day fatty acid intake relationships were also examined.

**Results:**

Cinnamon did not change gastric emptying parameters, postprandial triacylglycerol or glucose concentrations, oxidative stress, arterial function or appetite (p < 0.05). Strong relationships were evident (p < 0.05) between GE T_half _and 1-day palmitoleic acid (r = -0.78), eiconsenoic acid (r = -0.84) and total omega-3 intake (r = -0.72). The ingestion of 3 g cinnamon had no effect on GE, arterial stiffness and oxidative stress following a HF meal.

**Conclusions:**

3 g cinnamon did not alter the postprandial response to a high-fat test meal. We find no evidence to support the use of 3 g cinnamon supplementation for the prevention or treatment of metabolic disease. Dietary fatty acid intake requires consideration in future gastrointestinal studies.

**Trial registration:**

Trial registration number: at http://www.clinicaltrial.gov: NCT01350284

## Background

Free-living individuals are in the postprandial hyper-triglyceridemic state for the majority of a 24-h period [1]. Dietary supplementations, such as traditional spices, that can limit lipemia and glycemia in the fed state, have important implications for prevention and management of metabolic diseases. Two decades ago, cinnamon (*Cinnamomum zeylanicum*) was proposed as a treatment for type 2 diabetes (T2D) when it was shown to display insulin-mimetic properties [2]. Cinnamon has been proposed to act on numerous mechanisms relating to glucose and insulin function including improved cellular uptake of glucose through stimulation of insulin receptor kinase activity, increased insulin receptor phosphorylation and glycogen synthesis activity and reducing inflammation through antioxidant effects [3-7]. Chronic supplementation of 1 to 6 g cinnamon has been shown to have similar effects with regards to lowering of fasting glucose and lipid levels in diabetic patients [8]. Acutely, a 5 g cinnamon bolus improved glycemic responses and insulin sensitivity when given 12 hours prior to, or with, an oral glucose tolerance test (OGTT) in healthy adults [9].

In addition to rate of glucose removal from circulation, plasma glucose concentration is also determined by the rate of glucose entering the circulation [10]. Gastric emptying (GE) is an important determinant of rate of glucose appearance and blood glucose homeostasis in healthy and diabetic populations [11,12]. Delayed GE could be part of the mechanism by which cinnamon improves glucose tolerance. When combined with a semi-solid, low-fat meal, 6 g cinnamon reduced postprandial glycemia and delayed GE in healthy subjects [13].

Vascular dysfunction has emerged as a critical step in the development and progression of CVD, specifically atherosclerosis [14]. Vascular dysfunction refers to impairments in nitric oxide (NO)-mediated endothelium-dependent dilation, which is inversely related to an increase in vessel stiffness [15]. These impairments may stem from decreased NO synthesis and/or release, in combination with exaggerated consumption by reactive oxygen species (ROS) [16]. It has been postulated that postprandial lipemia may promote vascular dysfunction via an oxidative stress pathway [17-19].

The actions of dietary fatty acids on the gastrointestinal (GI) tract are still poorly understood. Acute ingestion of dietary fat can potently delay GE and reduce appetite [20]; these effects are partly mediated by the secretion of GI peptides including glucagon-like peptide-1 (GLP-1) and cholecystokinin (CCK) [21]. Dietary fatty acid intake may be one factor that affects inter- and intra- subject variability in GE rates.

We aimed to determine if acute supplementation of 3 g cinnamon would reduce postprandial glycemic and lipemic responses to a high-fat (HF) meal through a delay in GE or an alternative mechanism. We also evaluated the effect of cinnamon ingestion on oxidative stress, vascular function, appetite sensations and subsequent food intake in healthy subjects. Additionally, we examined the relationship between previous day fatty acid intake and GE of the HF test meal.

## Methods

### Subjects

Nine apparently healthy subjects (3 male, 6 female; age 26.2 ± 3 years; mass 66.5 ± 11.3 kg; BMI 22.4 ± 2.5 kg/m^2^; body fat 22.2 ± 6.5%) consented to participate in the study, which was approved by the Local Research Ethics Committee. Subjects had no history of GI-related complaints, CVD or diabetes and were not currently taking antioxidant or lipid-lowering medication. Subjects were non-smokers and were recreationally active (performing < 60 min of moderate to intense exercise per day, on every day of the week) determined using a 3 month exercise questionnaire. Fasting blood lipid, glucose and blood pressure (BP) levels were all within the normal limits. Subjects were required to rate at least 50% of the food items that would be presented in the buffet meal as 5 or higher in a food preference questionnaire for study inclusion. Two subjects were identified as restrained eaters (scoring > 12 on the eating restraint section (factor 1) of the Three-Factor Eating Questionnaire [22]).

### Experimental design

Each subject completed two separate 1-day trials separated by 28 days: cinnamon and placebo (wheat flour) supplementation. Trials were conducted in a single-blind, placebo controlled randomized fashion and were identical apart from the content of the capsules. During the 3 d before the first trial, subjects recorded all of the food and drink that they consumed and repeated this diet before the second trial. Energy, macronutrient and fatty acid intake were calculated using CompEat Pro Nutritional Analysis software (Version 5.8; Nutrition Systems, Grantham, UK). Physical activity was also logged during this time period. Subjects were instructed to minimize their consumption of foods with naturally high ^13^C abundance on the day preceding each trial and were asked to refrain from alcohol consumption and vigorous exercise. Apart from these requirements, subjects ate and exercised *ad libitum *between the two testing periods.

### Study day protocol

Subjects reported to the laboratory after a 12 h overnight fast. Following 15 min interval of supine rest, measurements of arterial stiffness (see measurements) and BP were obtained and a baseline blood sample was collected. Subjects returned to the seated position and after a 10 min interval, baseline breath samples were taken and visual analogue scale (VAS) questionnaires were completed (*t *= -15 and -10 min). Once baseline measurements were taken, subjects consumed the test meal within 15 min (see below). Upon completion of the meal (*t *= 0 min), sequential postprandial measurements of GE, appetite sensations, arterial stiffness, BP and plasma glucose and lipids responses were taken. Expired breath samples were collected at *t *= 0 min, every 5 min for the first half hour after meal consumption and thereafter in 15-min intervals from *t *= 30 until 360 min for the detection of ^13^CO_2 _(see measurements). VAS questionnaires were administered after consumption of breakfast (*t *= 0 min) and every 30 min until *t *= 360 min. Arterial stiffness, BP measurements and blood samples were collected hourly from *t *= 60 to *t *= 270 min (*t *= 240 min for blood samples). At the end of postprandial assessments (*t *= 360 min), subjects were presented with an assortment of cold lunch-type buffet foods. The meal comprised of six slices of wholegrain bread (235 g), six slices of white bread (235 g), 115 g sliced ham, 115 g sliced chicken, 100 g grated cheddar cheese, 60 g lettuce, 125 g cherry tomatoes, 80 g sliced cucumber, 90 g sweet corn, 100 g coleslaw, 30 g butter, 30 g mayonnaise, 30 g relish, 415 g strawberry yoghurt, 6 biscuits (75 g), 120 g chocolate Swiss roll, 60 g crisps and 300 ml water. The total energy content of the buffet meal was 16504 kJ. The selection presented was in excess of anticipated consumption. Subjects were given 30 min (i.e. *t *= 360 - 390 min) to eat *ad libitum *until 'comfortably full'. After ingestion of the buffet meal, subjects completed another VAS questionnaire and were then free to leave the laboratory. Additional VAS questionnaires were administered after consumption of the test meal and buffet meal (*t *= 0 and 390 min) to evaluate meal palatability as well as sensations of nausea and well-being. They recorded their subsequent food intake for the remainder of the test day.

### Test meal

The test meal consisted of three pancakes (36 g flour, 44 g egg, 70 g whole milk, 30 g sunflower oil) served with 20 g chocolate spread and 300 ml of water. The test meal was enriched with 150 μl ^13^C octanoic acid (Cambridge Isotope Laboratories, Andover, MA, USA), which was solubilized in the egg yolk prior to cooking. After homogenizing the yolk, it was mixed with the other ingredients to ensure uniform distribution of the label throughout the pancake batch. The test meal provided 2646 kJ of energy and consisted of 42 g carbohydrate, 46 g fat and 13 g protein. Subjects were instructed to ingest 8 gelatin capsules (four directly before and after meal) totaling 3 g Cinnamon powdered spice (*Cinnamomum zeylanicum*; Schwartz, UK) or a wheat flour placebo (Odlums, Ireland).

### Measurements

#### Gastric emptying

Gastric emptying was determined using the ^13^C octanoic acid breath test [23,24]. Breath samples were collected into 10 ml tubes (Exetainers; Labco, Bucks, UK). The analysis of the ^13^CO_2_/^12^CO_2 _enrichment in breath samples was performed using an ABCA (Europa Scientific, Crewe, UK) isotope ratio mass spectrometer. GE parameters, gastric half emptying time (*T*_half_), lag phase (*T*_lag_), latency time (*T*_lat_) and ascension time (*T*_asc_) were calculated using previously described methods [23,25]. Cumulative excretion of ^13^CO_2 _(as a percent of ingested dose) was also calculated.

#### Arterial stiffness

Arterial stiffness was measured using the PulseTrace PCA 2 device (Micro Medical, Kent, UK). The device measures the digital volume pulse (DVP) through the use of a photoplethysmographic transducer placed on the index finger of the right hand, transmitting infra-red light at 940 nm. The device permits the calculation of the stiffness index (SI; m/s) and the reflection index (RI; %) [26,27]. Heart rate (HR) in beats/min (bpm) was also measured by transmission of the DVP.

#### Blood pressure

Systemic arterial blood pressure (BP) was measured at the brachial artery using an Omran M5-I fully automatic BP monitor (Surrey, UK). Measurements were taken in triplicate directly after arterial stiffness measurements in the supine position and an average of these readings was recorded.

#### Blood analyses

Blood samples were collected into K-EDTA and NaF tubes and placed on ice. Serum separating clot activator tubes were allowed to clot at room temperature. All samples were separated within 30 min of collection and stored at -70°C until analysis. Plasma glucose was determined by an immobilized enzyme membrane method in conjunction with a Clark electrode on a YSI 2300 analyzer (Yellow Springs, USA). Total cholesterol, TAGs and HDL were measured by enzyme assay kits, using an automated analyzer (Aeroset ™, Abbott Labs, USA [28]). LDL cholesterol was calculated using the Friedewald equation [29]. All samples for each subject were analyzed in a single analyzer run. CVs were < 7.7% for glucose and < 10.0% for all blood lipids.

#### Serum lipid hydroperoxides

Serum lipid hydroperoxides (LOOHs) (as measures of lipid peroxidation) were incubated with FOX-1 assay and quantified using a Shimadzu UV-VIS 1240 Spectrophotometer, (Mason Technology, Belfast, Northern Ireland [28]. CV was < 16% for LOOH.

#### Appetite sensations and subsequent energy intake

Sensations of hunger, desire to eat, fullness, thirst, tiredness and coldness were assessed using a VAS questionnaire [30]. Although it was important to examine general well being, the latter sensations were primarily included so that subjects were unaware that our primary outcome measures were sensations of appetite. Each VAS assessed a sensation on a 150 mm horizontal line anchored at the beginning and end by opposing statements.

The quantity of food consumed in the buffet meal was recorded to the nearest 0.1 g and the total energy (kJ) and macronutrient intakes (% of energy) were subsequently calculated. Time taken to complete the buffet meal completion (in min) was also recorded.

### Statistics

Data were checked for normal distribution before statistical analysis was preformed. Appetite sensation data was transformed by natural log. Fasting measurements of arterial stiffness, blood pressure and plasma lipid and glucose concentrations were calculated as the mean of the values collected at baseline (~*t *= -30 to -20 min). Baseline GE and appetite sensation scores were calculated as the mean of values collected at *t *= -15 and -10 min. Blood biomarkers, appetite sensations and arterial stiffness data were analyzed using a two-way (time × supplement) repeated-measures ANOVA. Incremental area under the glycemic curve (IAUC) was calculated using the trapezoidal rule by subtracting baseline values from measured plasma glucose concentrations. Relationships between variables were compared using Pearson correlations. Paired-sample *t *tests were used to compare GE parameters, habitual diet, physical activity intensity and duration as well as food intake at the buffet meal (quantity, energy consumed and macronutrient distribution). Statistical significance was established at the p < 0.05 level and the mean values ± SD are reported. All statistical analyses were carried out using SPSS-version 16.0 (SPSS, Inc., Chicago, IL, USA). Power calculation was conducted for gastric emptying T_half _the primary endpoint evaluation. A sample size of 9 subjects was necessary to detect a 15.8% change in GE rate [13] in a two-sided paired Students' t-test with alpha set at 5% and a power of 80%. Trial registration number: at http://www.clinicaltrial.gov: NCT01350284.

## Results

Test days were well tolerated by all subjects. Subjects successfully repeated their food and physical activity diaries as indicated by no significant differences in energy or macronutrient intake (*p *> 0.05) and in total and exercise intensities respectively (*p *> 0.05) for 3 days prior to each treatment. Usual dietary intake was consistent with guidelines for healthy living (51% of energy from carbohydrate, 31% fat, and 18% protein).

### Gastric Emptying

No significant effect of cinnamon supplementation was observed on gastric emptying parameters (Table 1).

**Table 1:** Mean values for gastric emptying parameters and food intake from the buffet meal after the ingestion of high-fat meal supplemented with 3 g wheat flour (placebo) or cinnamon

Variable	Placebo	Cinnamon
Gastric emptying		
*T*_half _(min)	237 ± 32	245 ± 49
*T*_lag _(min)	136 ± 12	143 ± 22
*T*_lat _(min)	40 ± 6	43 ± 9
*T*_asc _(min)	197 ± 35	202 ± 48
Cumulative excretion of^13^CO_2 _(%)	55.1 ± 10.0	56.3 ± 4.9
Food intake		
Energy intake (kJ)	3056 ± 1130	3324 ± 1516
Quantity consumed (g)	420 ± 200	467 ± 151
Carbohydrate (%)	40 ± 13	43 ± 12
Fat (%)	40 ± 8	36 ± 10
Protein (%)	21 ± 10	22 ± 9
Time (min)	15 ± 3	15 ± 5

*T*_half_, gastric half emptying time; *t*_lag_, lag phase; *t*_asc_, ascension time; *t*_lat_, latency time.

Data are means ± SD, n = 9.

No significant differences were found between treatments, p > 0.05.

### Cardiovascular Measures

#### Arterial stiffness

There were no mean differences between or within the groups for SI (time × supplement interaction, p > 0.05) (Table 2). However, a main effect for time was observed whereby SI decreased postprandially in both supplements (pooled placebo and drug data, p < 0.05). There were no reported differences either between or within supplements for RI over time following the ingestion of the test meal (time × supplement interaction, p > 0.05) (Table 2).

**Table 2:** Mean stiffness and reflection index values after the ingestion of high-fat meal supplemented with 3 g wheat flour (placebo) or cinnamon

Variable	Placebo	Cinnamon
Stiffness Index (m/sec)		
Baseline	5.58 ± 0.55	5.78 ± 0.48
60 min	5.53 ± 0.49	5.46 ± 0.51
120 min	5.42 ± 0.44	5.32 ± 0.46
180 min	5.39 ± 0.41	5.34 ± 0.71
240 min	5.34 ± 0.32	5.31 ± 0.36
300 min*	5.47 ± 0.45	5.19 ± 0.41
Reflection Index (%)		
Baseline	68.92 ± 18.15	68.72 ± 21.42
60 min	61.26 ± 12.73	60.15 ± 12.19
120 min	61.64 ± 8.79	63.76 ± 8.92
180 min	66.74 ± 12.63	69.37 ± 12.49
240 min	71.57 ± 15.84	69.70 ± 8.88
300 min	69.78 ± 12.99	68.68 ± 12.71

Data are means ± SD, n = 9.

No significant differences were found between treatments, p > 0.05. * Main effect for time for Stiffness Index; pooled placebo and drug data, p < 0.05

#### Blood pressure and heart rate

There were no differences either between or within the groups for both systolic and diastolic BP over time following the ingestion of the test meal (time × supplement interaction, *p *> 0.05). There were no mean differences between or within the groups for HR (time × supplement interaction, *p *> 0.05). However, a main effect for time was observed whereby HR decreased over time across both trials (60 ± 10 bpm at baseline vs. 52 ± 9 bpm at 300 min post meal in placebo trial; 61 ± 12 bpm at baseline vs. 51 ± 9 bpm at 300 min post meal in cinnamon trial; pooled placebo and drug data, *p *< 0.05).

### Blood biomarkers

#### Plasma glucose

**Figure 1:**
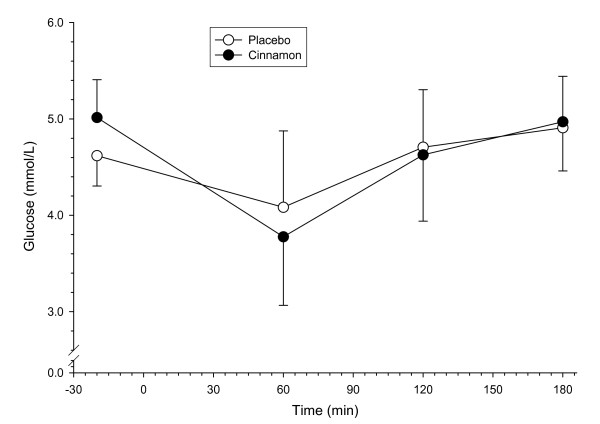
**Plasma glucose concentrations in healthy subjects after the ingestion of a high-fat test meal supplemented with 3 g cinnamon or placebo**. Values are means, with standard deviations represented by vertical bars (n = 8).

Baseline plasma glucose concentrations tended to be higher after cinnamon supplementation compared to placebo (p > 0.05; n = 8 for all blood derived measurements). Plasma glucose concentrations changed across time in response to both supplementations (p < 0.0001) (Figure 1). There was no significant interaction between time and supplement (p > 0.05). Three hours after meal ingestion all plasma glucose concentrations were similar to baseline concentrations, regardless of supplement. IAUC following placebo (-22.2 ± 87.6 mmol/l/min) was similar to that following cinnamon ingestion (-14.2 ± 29.1 mmol/l/min, p = 0.819).

#### Plasma lipids

Baseline plasma TAG, total cholesterol, HDL and LDL concentrations did not differ significantly between trials (Table 3). There were no changes in TAG between trials (time × treatment interaction, p > 0.05) but there was a main effect for time (pooled placebo and cinnamon data, p < 0.05). There were no changes in plasma total cholesterol, HDL, or LDL levels either within or between the trials (time × supplement interaction; p > 0.05).

**Table 3:** Mean plasma lipid values after the ingestion of high-fat meal supplemented with 3 g wheat flour (placebo) or cinnamon

Variable	Placebo	Cinnamon
TAG (mmol/l)		
60 min	0.84 ± 0.27	0.80 ± 0.32
120 min	0.93 ± 0.36	0.88 ± 0.32
180 min	1.01 ± 0.36	0.95 ± 0.37
Total Cholesterol (mmol/l)		
60 min	3.80 ± 0.59	3.93 ± 0.43
120 min	3.79 ± 0.55	3.77 ± 0.68
180 min	3.85 ± 0.52	3.78 ± 0.69
HDL (mmol/l)		
60 min	1.45 ± 0.31	1.49 ± 0.41
120 min	1.39 ± 0.27	1.46 ± 0.38
180 min	1.45 ± 0.32	1.45 ± 0.36
LDL (mmol/l)		
60 min	1.97 ± 0.57	2.07 ± 0.30
120 min	1.98 ± 0.54	1.91 ± 0.48
180 min	1.92 ± 0.46	1.90 ± 0.41

Data are means ± SD, n = 9.

No significant differences were found between treatments, p > 0.05.

#### Serum lipid hydroperoxides

**Figure 2:**
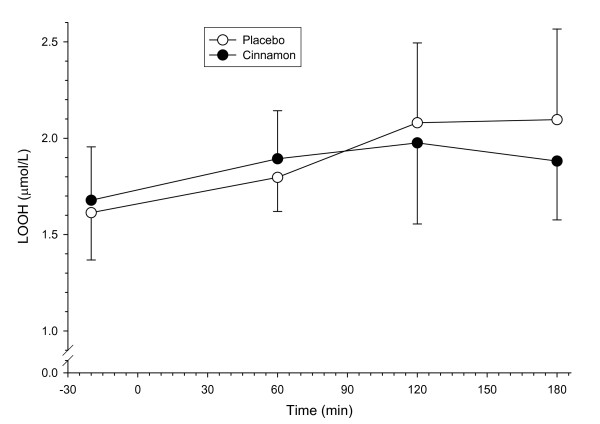
**Lipid hydroperoxides (LOOHs) concentrations in healthy subjects before and after the ingestion of a high-fat test meal supplemented with 3 g cinnamon or placebo**. Values are means, with SD represented by vertical bars (n = 8).

There was a main effect for time for LOOHs with levels increasing over time throughout the course of the trials (pooled placebo and cinnamon data, p < 0.05) but there were no changes in mean LOOHs between or within the groups (time × supplement interaction, p > 0.05) (Figure 2).

#### Appetite sensations and subsequent energy intake

**Figure 3:**
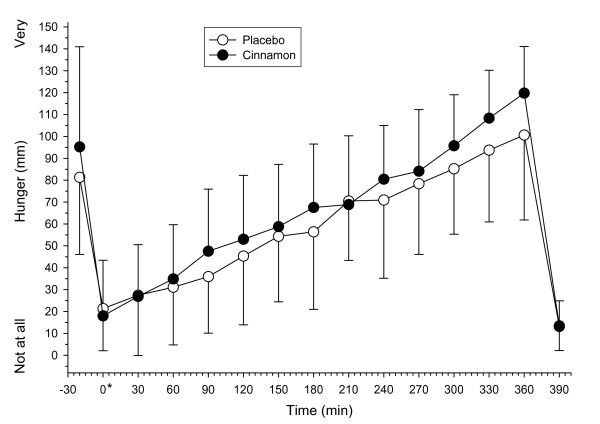
**Visual analogue scale (VAS) scores for sensations of hunger in healthy subjects before and after the ingestion of a high-fat test meal supplemented with 3 g cinnamon or placebo (n = 8)**. * Represents the time immediately after breakfast. Values are means, with SD represented by vertical bars.

The variables thirst, tiredness and coldness did not vary significantly with supplementation or over time (data not shown). Baseline appetite sensation VAS scores did not differ significantly between conditions. Changes to appetite sensation scores were evident over time after ingestion of test meals (p < 0.05). No significant effect of treatment was observed on sensations of hunger (Figure 3), desire to eat and fullness (data not shown). No differences between sensations of palatability, pleasantness, nausea and stomach pain recorded when treatments were compared after the test breakfast (*t *= 0 min) and buffet-style lunch meal (*t *= 390 min). No significant effect of treatment was observed for quantity of food consumed, energy intake, percentage of energy provided by carbohydrate, fat or protein and time taken to consume the buffet meal (Table 1).

#### Relationship between variables

Significant positive correlations were observed between TAG levels and LOOHs (r = 0.52, p < 0.0001) and SI and LOOH (r = 0.24, p < 0.05). GE T_half _was inversely related to energy intake in the buffet meal after cinnamon ingestion (r = -0.67, p < 0.05) but not in the placebo trial (r = -0.62, p > 0.05).

#### Relationship between gastric emptying and 3-day background fatty acid intake

No significant differences were observed between 1-day background fatty acid intake before cinnamon and placebo trials. 1-day intake of palmitoleic acid (C16:1, r = -0.78, p < 0.05), eiconsenoic acid (C20:1, r = -0.84, p < 0.01) and total *n*-3 intake respectively (r = -0.72, p < 0.05) were inversely related to GE T_half _of the placebo-supplemented HF meal (Table 4). 1-day intake of C16:1, C20:1 and total n-3 fatty acids accounted for 61, 71 and 51% of the variance in GE T_half _respectively. No significant relationships between GE T_half _after cinnamon ingestion and 1-day of palmitoleic acid, eiconsenoic acid or total n-3 intake were observed (p > 0.05).

**Table 4:** Relationship between previous day reported intake of fatty acids (1-day) and GE T_half _of high-fat meal supplemented with 3 g wheat flour (placebo) or cinnamon (n *= *9)

	Placebo	r	Cinnamon	r
Energy (kJ)	7963 ± 871	-0.55	8210 ± 1599	0.08
Fat (g)	63.46 ± 12.39	-0.22	72.96 ± 24.67	0.43
16: 1 (g)	0.29 ± 0.17	-0.78 †	0.31 ± 0.14	-0.53
20: 1 (g)	0.11 ± 0.12	-0.84 ††	0.15 ± 0.18	-0.29
Total *n*-3 (g)	0.48 ± 0.37	-0.72 †	0.55 ± 0.40	-0.54

*T*_half_, gastric half emptying time; C16:1, palmitoleic acid; C18:1n-9, oleic acid; C20:1,eiconsenoic acid; total n-3, total omega-3 intake.

Data are means ± SD, n = 9.

Correlations were significantly different: † = p < 0.05, †† = p < 0.01.

## Discussion

The current study tested the hypothesis that supplementing a single HF breakfast with 3 g of cinnamon would delay GE of a high-fat solid meal utilizing the ^13^C octanoic acid breath test, and consequently reduce postprandial blood glucose and lipid concentrations.

### Gastric Emptying, Metabolic Variables and Appetite

We were unable to induce significant changes in GE using 3 g of cinnamon. The test meal (65% of energy from fat) was mainly from sunflower oil, which contains approximately 70% linoleic acid (C18:2*n*-6, a PUFA [31]). Long-chain fatty acids have a potent inhibitory effect on GE rate [20,32] and have also been shown to increase CCK and GLP-1 concentrations [21]. We propose that cinnamon does not delay GE over and above the effects of the fat content of the meal. Furthermore, we found similar postprandial glycemic and lipemic responses under both conditions. However, it should be noted that we were unable to measure a hyperglycemic or hyperlipidemic state. Studies which employed the largest doses of cinnamon relative to carbohydrate in the test meal (carbohydrate/cinnamon ratio of 15 or lower [9,13]) appear to have had the most potent effects on reducing postprandial glycemia [33]. In spite of the current high ratio of 14, we did not achieve a significant blood glucose-lowering effect. This is possibly due to glucose absorption from the small intestine being affected by the fat content of a meal [34].

Recent data indicates that the addition of 3 g cinnamon to a low-fat rice pudding test meal had no significant effect on GE rate or postprandial glycemia in healthy individuals [35]. However, cinnamon did significantly lower serum insulin levels and increase GLP-1 concentrations, a GI peptide which has been shown to increase glucose-dependent secretion of insulin, delay GE and reduce glucose absorption and postprandial glycemia [36,37]. When added to the same test meal, 6 g cinnamon significantly delayed GE and reduced postprandial glycemia but the decrease in blood glucose concentration was more apparent than the delay in GE rate suggesting that GE cannot be the sole mechanism explaining lower blood glucose responses following cinnamon ingestion [13]. Agreeing with the findings of others [13,33,34], we found that cinnamon did not influence appetite sensations or subsequent food intake, probably as a result of similar GE rates between conditions [38]. Together with data presented in the current study, cinnamon is unlikely to be relevant in affecting the postprandial response to HF meals.

### Diet and fatty acid intake

Assessment of previous day dietary intake indicated that a higher intake of C16:1, C20:1 and total *n-3 *was associated with a shorter GE *T*_half _of the HF meal supplemented with the wheat flour placebo. To our knowledge, this is the first observation of specific dietary fatty acids from preceding diet affecting GI transit in humans. A single meal, supplemented with *n*-3 PUFAs, was less capable of triggering GLP-1 and CCK compared to other fats, resulting in a more rapid GE of a HF breakfast [39] while others showed that *n*-3 PUFA fish oil reduced CCK release and gallbladder contraction without affecting GE [40]. Both GLP-1 and CCK are putative mediators of the ileal brake, a feedback mechanism responsible for delaying transit and facilitating digestion, in response to lipids in the distal GI tract [41]. Our current findings extend these observations to illustrate that even short-term intake of *n-3 *fatty acids is associated with faster GE rates, in a population who were not eating a HF diet. This means that mechanisms apart from acute release of GLP-1 and CCK, must mediate the effects of specific fatty acids on GI transit. Recently, a 3-day HF yoghurt supplementation, rich in C18:2*n*-6 accelerated the GE rate of a test meal rich in the same fatty acid [42]. It is interesting to note that background intake of C18:2*n*-6, which was high in the test meal, did not show a strong association with GE of the meal. Our observations suggest that GE *T*_half _of a HF meal is not just specifically affected by a background intake of that specific fatty acid and that the process of adaptation to a HF diet may involve mechanisms other than desensitization to a specific fatty acid. It is likely that different adaptations are continuously taking place in the gut, in response to the balance of fatty acids in the diet. It is tempting to speculate about the potential mechanisms for fat sensing and adaptation following the recently sequenced GPR120 protein, expressed on intestinal cells, and demonstrated to be differentially sensitive to different fatty acids [43].

### Vascular Function and antioxidant capacity

One of the principle findings of this study was that no changes were observed in relation to the measures of vascular function. Interestingly, there was a main effect for time indicating a decrease in SI which at face value appears paradoxical given the transient impairment in vessel function following the ingestion of a HF meal [44,45]. This apparent contradiction might be explained by the fact the test meal we used may not have been of sufficient energy and, in particular, fat content to evoke a change in vessel function. In most of the related literature the postprandial TAG concentration associated with vascular dysfunction (~2.0 mmol/l; [17,18,44]) is double than that presently observed. Moreover, the HF meal in the current study contained 297 - 1742 kJ less energy (and 14 - 34 g less fat) than other similarly designed investigations [17,45-47]. Such discrepancies in meal composition further highlight the need for a standardized, physiologically relevant HF meal [48] to be used in future corresponding studies, similar to the OGTT.

Due to its polyphenolic nature, cinnamon is thought to exhibit antioxidant properties [3,49] which may be anti-atherogenic. It is proposed that the impairments in blood vessel function following the ingestion of HF loads are perpetrated via an oxidative stress mechanism that can increase the unwanted consumption of NO and favor the formation of further ROS, such as peroxynitrite (ONOO^-^) [17-19,28,44]. In the present study cinnamon ingestion had no apparent effect on indices of oxidative stress as a main effect for time was observed for LOOHs.

It has been documented that even high-normal fasting glucose levels can aggravate arterial stiffness [50] but when cinnamon was added to rats fed a high-fat high-fructose diet hepatic glycogen, hepatic insulin receptors and glut4 transporter in muscle tissue all increased [51]. Despite no reported change in arterial stiffness using our physiologically relevant high-fat meal, the possibility therefore exists that cinnamon could modulate stiffness by affecting (hepatic) glycemic control and thus this relationship merits further scrutiny.

Emerging research postulates the existence of a diurnal variation in endothelial function [52]. Fluctuations in the competitive balance between intrinsic local vasodilator function and sympathetic nervous system (SNS) α-adrenoreceptor-mediated vasoconstriction have recently been proposed as one potential mechanism to explain such findings [53]. Given the conceptual relationship between endothelial function and arterial stiffness, and the observed main effect for a decrease in HR (as an indirect measure of SNS activity), it is tempting to speculate that this explanation may in some way account for the SI data in the current investigation. Conversely, this was tempered by the fact that no changes in RI were recorded.

This study may have been underpowered and therefore a small effect below the detection threshold of the study cannot be ruled out. Three grams of cinnamon was used as because it was shown to have a similar chronic effect on fasting serum glucose and lipid profiles as 6 g [8]. However, recent evidence suggests a dose-dependent relationship for cinnamon consumed and the delay in GE [13,35].

## Conclusions

We found no evidence for delayed GE rate or reduced indices of oxidative stress in response to a HF meal supplemented with 3 g cinnamon compared to placebo. Cinnamon did not change postprandial glycemic and lipemic responses, arterial stiffness or appetite. Given the association between 1-day fatty acid intake and GE *T*_half_, we suggest that controlling for background fatty acid composition requires consideration in future gastrointestinal studies.

## List of abbreviations

GE: gastric emptying; T2D: type 2 diabetes; CVD: cardiovascular disease; HF: high-fat; OGTT: oral glucose tolerance test; NO: nitric oxide; ROS: reactive oxygen species; GI: gastrointestinal; GLP-1: glucagon-like peptide-1; CCK: cholecystokinin; BP: blood pressure; VAS: visual analogue scale; *T*_half_: gastric half emptying time; *T*_lag_: lag phase; *T*_lat_: latency time; *T*_asc_: ascension time; DVP: digital volume pulse; SI: stiffness index; RI: reflection index; LOOH: lipid hydroperoxides; SNS: sympathetic nervous system

## Competing interests

The authors declare that they have no competing interests.

## Authors' contributions

The author's responsibilities were as follows - OM and AS contributed to all aspects of this study. CM, PM, GWD, TT and ED contributed to acquisition of data, or analysis and interpretation of data. All authors read, revised and approved the final manuscript.
